# Effectiveness of Body Weight-Supported Gait Training on Gait and Balance for Motor-Incomplete Spinal Cord Injuries: A Systematic Review with Meta-Analysis

**DOI:** 10.3390/jcm13041105

**Published:** 2024-02-15

**Authors:** Rubén Arroyo-Fernández, Raquel Menchero-Sánchez, Diana P. Pozuelo-Carrascosa, Helena Romay-Barrero, Araceli Fernández-Maestra, Inés Martínez-Galán

**Affiliations:** 1 Faculty of Physical Therapy and Nursing, University of Castilla-La Mancha, 45071 Toledo, Spain; ruben.arroyo@uclm.es (R.A.-F.); raquel.menchero@uclm.es (R.M.-S.); ines.martinez@uclm.es (I.M.-G.); 2 Water and Health Research Group (GIAS), University of Castilla-La Mancha, 45004 Toledo, Spain; 3 Department of Physical Medicine and Rehabilitation, Hospital General Universitario Nuestra Señora del Prado, 45600 Talavera de la Reina, Spain; 4 Faculty of Nursing, University of Castilla-La Mancha, 16071 Cuenca, Spain; dianap.pozuelo@uclm.es; 5 Department of Physical Medicine and Rehabilitation, National Hospital for Paraplegics, 45004 Toledo, Spain; araceli.fernandez@uclm.es

**Keywords:** spinal cord injury, body weight-supported, gait, balance, rehabilitation

## Abstract

Objective. This review aims to analyse the effectiveness of body weight-supported gait training for improving gait and balance in patients with motor-incomplete spinal cord injuries. Method. Relevant articles were systematically searched in electronic databases to identify randomised controlled trials of body weight-supported gait training (either with methods of robotic, manual, and functional electrical stimulation assistance) versus conventional physical therapy or no intervention. Subjects were >16 years-old with motor-incomplete spinal cord injury (AIS C or D). Primary outcomes were gait-related parameters (functionality, endurance, and speed) and balance. Quality of life was included as a secondary outcome. Articles were selected up to 31 December 2023. Results. Fifteen studies met the inclusion criteria (n = 673). Nine studies used robotic assistance, four trials performed manual assistance, one study functional electrical stimulation assistance, and one trial performed the intervention without guidance. Robot-assisted body weight-supported gait training improved walking functionality (SMD = 1.74, CI 95%: 1.09 to 2.39), walking endurance (MD = 26.59 m, CI 95% = 22.87 to 30.31), and balance (SMD = 0.63, CI 95% = 0.24 to 1.02). Conclusions. Body weight-supported gait training is not superior to conventional physiotherapy in gait and balance training in patients with motor-incomplete spinal cord injury. However, body weight-supported gait training with robotic assistance does improve walking functionality, walking endurance, and balance, but not walking speed.

## 1. Introduction

Spinal cord injury is a serious medical condition, which often results in severe morbidity and permanent disability [1]. Damage to the descending and ascending tracts results in impairment of the postural control system, which affects standing, locomotion, and voluntary movement [2,3]. Approximately half of spinal cord injuries are classified as motor-incomplete spinal cord injury (motor-iSCI) [4,5] and >75% of patients suffering from this condition regain some form of ambulatory function [6].

The partial preservation of descending supraspinal pathways [7,8] potentially enables the recovery of autonomous gait [3,9], a process where neuroplasticity plays a key role [10]. Therapeutic approaches for gait training focus on the ability of the central nervous system to modify neural pathways and synapsis, which leads to interventions based on the systematic execution of task-specific training [11]. Gait training programs for people with spinal cord injury have evolved in recent decades, from manually assisted overground training or body weight-supported gait training (BWSGT) to robotic-assisted body weight-supported gait training (RAGT) [12]. BWSGT involves the practice of stepping on a motorized treadmill while unloading a percentage of a person’s body weight using a counterweight-harness system, either with assistance (e.g., manual, functional electrical stimulation (FES), robotic) or not [13]. Manual assistance easily leads to physical exhaustion of the therapist, while FES assistance stimulates nerves and muscles using low-frequency electrical pulses to produce immediate functional activity [14]. Technological evolution has allowed the inclusion of RAGT devices (such as Lokomat^®^), reducing professional physical exhaustion and providing new results to rehabilitation [15]. Former meta-analyses [12,16,17] assessing the effectiveness of these forms of gait training either jointly included patients with complete and incomplete SCI or did not consider all forms of assistance previously described in BWSGT. However, recent studies suggest that, in the case of complete spinal cord injuries, robotic assistance could be more beneficial [18,19]. This review with meta-analysis focuses on motor-iSCI and widens the scope of assessed outcome variables by incorporating balance and quality of life.

The present systematic review and meta-analysis of randomised controlled trials aimed to summarize the evidence about the effects of BWSGT in subjects with motor-iSCI versus control, with a particular focus on gait parameters and balance as primary outcomes, as well as other clinical outcomes such as quality of life as a secondary outcome.

## 2. Materials and Methods

The design of this systematic review with meta-analysis followed the guidelines of the Preferred Reporting Items for Systematic Reviews and Meta-Analysis (PRISMA) Statement [20] and the recommendations of the Cochrane Collaboration [21]. Patient consent and approval from an ethics committee were not required to conduct the present study. The protocol was previously registered in the International Prospective Register of Systematic Reviews (PROSPERO) (registration number CRD42021269686).

### 2.1. Sources and Searches

Two independent researchers (RMS and RAF) conducted a systematic search in the following databases: MEDLINE (via PubMed), Scopus, Web of Science, Cochrane Central Register of Controlled Trials (CENTRAL), and Physiotherapy Evidence Database (PEDro). The following terms were employed for the search strategy: paraplegia, tetraplegia, quadriplegia, spinal cord injury, treadmill training, locomotion, body weight-supported, robot assisted, lokomat, balance, gait, and quality of life. Articles were selected up to 31 December 2023 without limitations in terms of language or gender. In addition, a manual reverse search was performed of the bibliographies in the included articles. See Supplementary Materials for the full electronic search strategy.

### 2.2. Study Selection 

After reviewing the title and abstract, two investigators (RMS and RAF) systematically evaluated the full texts of articles identified for eligibility, and a third investigator (DPPC) intervened to reach consensus in cases of disagreement. Studies had to meet the following inclusion criteria to be eligible for the systematic review: intervention—BWSGT (with or without guidance); participants—subjects greater than 16 years old with motor-iSCI (AIS C or D), either traumatic or non-traumatic SCI; study outcomes—gait (functionality, endurance, and speed) and balance as primary outcomes, and quality of life as a secondary outcome; study design—randomised controlled trials; comparator—other forms of conventional physical therapy (e.g., gait training without body-weight support, aerobic exercising, strength training, active/passive mobilizations) or no intervention at all. The exclusion criteria were intervention groups using powered exoskeletons with fully weight-bearing as a gait training method; studies with a quasi-experimental design or case-crossover studies; availability of only abstracts or conference presentations; not reporting data on the variables of interest. Studies involving AIS A or B subjects were included if they reported results separately with C and D subjects. In the case of duplicated studies, the most recent version or that with the data recorded closest to the end of treatment was included.

### 2.3. Data Extraction and Quality Assessment

Two researchers (IMG and HRB) performed the data extraction using a spreadsheet (Microsoft Excel, v.2021, Microsoft Corporation, Redmond, WA, USA) specifically designed for the present study. A third investigator (DPPC) compared both tables and presented the final data. Authors of the selected studies were contacted to obtain or clarify missing or unclear data if needed. Data available only in graphs were extracted using software for graph digitalization [WebPlotDigitizer v.4.7, Pacifica, CA, USA, https://apps.automeris.io/wpd/, accessed on 3 January 2024)].

For each study, the following data were extracted: the first author’s last name; year of publication; country; time since injury; age; level of lesion; sample size; type and duration of interventions; time points and outcome measures; and losses to follow-up.

The risk of bias was assessed based on recommendations by the Cochrane organization [21] using Review Manager [Review Manager (version 5.4.1), Copenhagen, Denmark) (RevMan). Two independent reviewers (RMS and RAF) evaluated the risk of bias and a third investigator (DPPC) resolved cases of disagreement. Six items were addressed for evaluating the risk of bias and the relevant risk was expressed in three levels (unclear, low, and high). Previously, the researchers had agreed that for the item “selective reporting”, studies without a registered protocol would be qualified as unclear or high risk depending on the final report.

### 2.4. Data Synthesis and Analysis

The inverse variance method was used for analysing all variables (gait characteristics and balance). Statistical heterogeneity was assessed using the I^2^ statistic, with 25%, 50%, and 75% representing low, moderate, and high heterogeneity, respectively [21]. Random effect and fixed effect analysis models were used when the I^2^ statistic was greater or lower than 50%, respectively. The mean difference (MD) was calculated for evaluating walking endurance and the standardised mean difference (SMD) was estimated for the assessment of walking functionality, walking speed, and balance. Confidence intervals were set at 95% (CI 95%) for all variables. The analysed results were those obtained in the earliest post-treatment evaluation for each of the included studies.

In addition to the global analyses, an analysis by subgroups was conducted for all variables to compare the methods of assistance in the BWSGT interventions (robotic, manual, FES, or none). Subsequently, an additional analysis by subgroups was performed to account for the time since injury (subacute (less than 12 months) or chronic (less than 12 months)).

Finally, publication bias was assessed visually by analysing funnel plots of gait-related parameters and balance.

All analyses were carried out using RevMan. The certainty of evidence was classified for each outcome as high, moderate, low, or very low following the Grades of Recommendation Assessment, Development and Evaluation (GRADE) method [22].

## 3. Results

**Figure 1:**
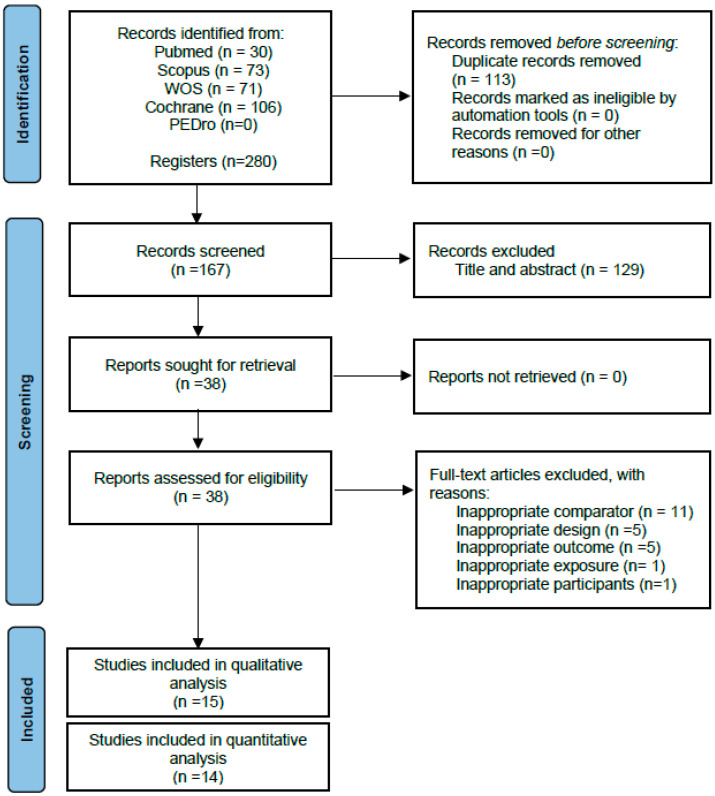
PRISMA flow diagram of the literature search and study selection.

Following the removal of duplicates, 167 articles were identified as eligible, of which 129 were eliminated after reading the title and abstract. Fifteen randomised controlled trials were included [23,24,25,26,27,28,29,30,31,32,33,34,35,36,37] in the qualitative synthesis after reading the full text. The study by Varoqui et al. [37] was finally excluded from the pooled quantitative analysis because of not reporting the results of the control group, yielding 14 randomised controlled trials that were included in the present meta-analysis (Figure 1).

The Supplementary Materials shows the reasons why studies were excluded after reading the full text. Additional information was requested from the authors of two studies [35,36,37] regarding outcome data, but no response was received.

### 3.1. Qualitative Summary of the Included Studies

Table 1 shows the characteristics of the included studies. Twelve studies [23,24,25,26,27,29,30,31,32,33,34,36] compared BWSGT to conventional physical therapy and three of them [28,35,37] to no treatment. All randomised controlled trials performed the BWSGT interventions over a treadmill, with no studies performing BWSGT overground. All randomised controlled trials included two treatment arms, with the exception of one study whose protocol involved three intervention groups [24]. The methods of assistance in the BWSGT interventions were robotic [23,25,28,29,32,33,35,36,37], manual [26,27,31,34], FES [30], or without guidance [24].

The sample size comprised a total of 673 subjects, 201 (30%) of which were women, and the average age ranged between 29 and 55 years. A total of 55 dropouts (8.17%) were identified, 32 in the BWSGT group and 23 in the control group. The intervention protocols showed heterogeneity in terms of overall duration of treatment (one to three months [23,24,27,28,31,34,35,36], four to six months [5,26,29,30,32,33] and frequency of sessions (two to three [23,24,27,29,30,32,34,36], five [25,26,28,31,35], and up to ten [33] sessions weekly).

The main variables assessed were: (a) walking functionality (measured via the scales Walking Index for Spinal Cord Injury [23,25,26,29,32,36], Spinal Cord Independence Measure III [25,30,32,36], and Functional Independence Measure-Locomotor section [23,27,29,30]); (b) walking speed (measured via the 10 m walk test [23,24,28,29,30,33,34,35,37], 50-foot walk test [27], or using instrumental devices [25,31]); (c) walking endurance (measured via the 6 min walk test [23,27,28,29,30,33,34,37]); and (d) balance (measured with the Timed up and Go Test [28,30,35,37], Berg Balance Scale [26,33,34], Tinetti test [24], or the modified Functional Reach Test [33,34]). Quality of life was evaluated in two studies using the Multiple Sclerosis Quality of Life-54 Instrument [26], Satisfaction with Abilities and Well-Being Scale [24], and the Short Form 36 Health Survey [24]. In terms of follow-up periods, nine studies [23,24,25,29,31,33,34,35,37] conducted only post-treatment evaluations, three randomised controlled trials [28,32,36] performed a short-term follow-up (1–3 months), and three trials [26,27,30] included medium-/long-term follow-ups (6–12 months).

### 3.2. Risk of Bias in the Included Studies

**Figure 2:**
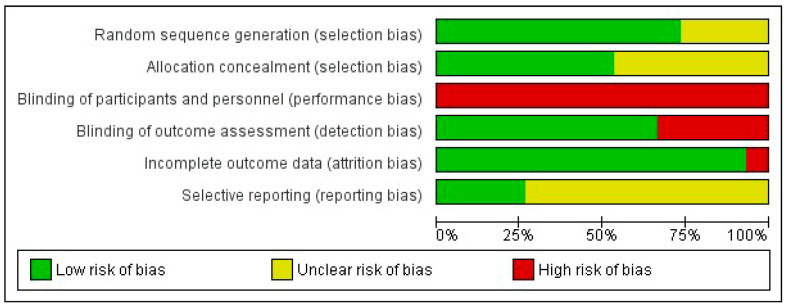
Risk of bias graph. The percentage (%) shows the risk of bias for each methodological domain of the tool.

The two researchers in charge of assessing the risk of bias agreed upon 82.7% of the items. Supplementary File S1 shows a detailed analysis of the risk of bias in the 15 included articles. The domain with the highest risk of bias was performance bias due to the impossibility of blinding both the subjects and researchers who applied the therapy in all the included studies. In terms of selection bias, low risk was observed in most randomised controlled trials (73.3%) for the random sequence generation item and in approximately half of the studies (53.3%) for the allocation concealment item, the rest being classified as unclear. Similarly, low risk was observed in ten studies (66.7%) for detection bias and in fourteen (93.3%) for attrition bias. Finally, eleven studies (73.3%) were rated as unclear in terms of reporting bias since they did not previously publish their research protocol. Figure 2 shows a summary of the five domains evaluated. Eight (53.3%) randomised controlled trials [23,24,25,29,30,31,33,34] presented moderate risk of bias with 4–5 items classified as low risk.

### 3.3. Quantitative Summary: Effects of Body Weight-Supported Gait Training

#### 3.3.1. Walking Functionality

**Figure 3:**
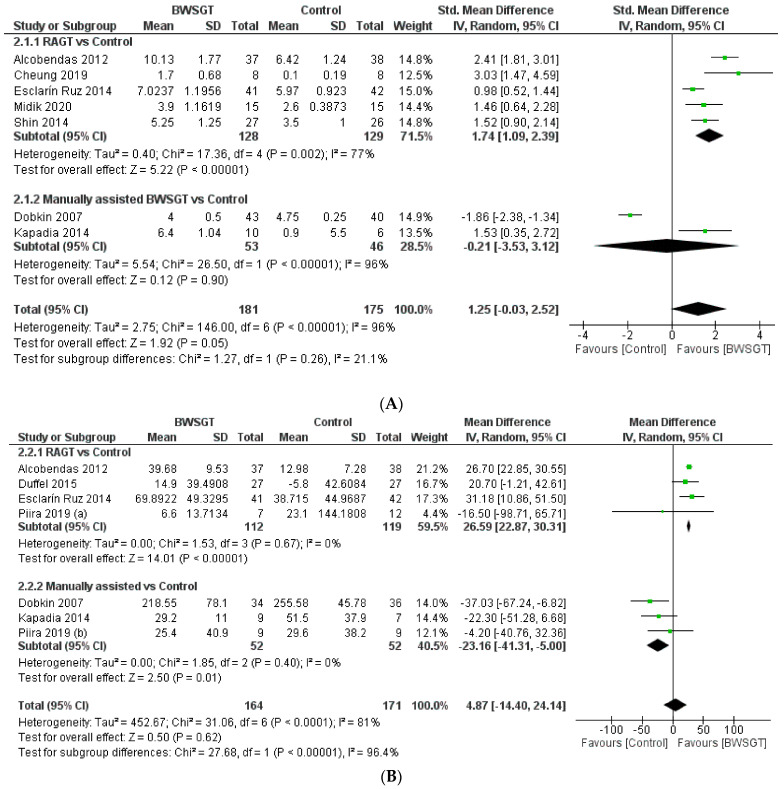

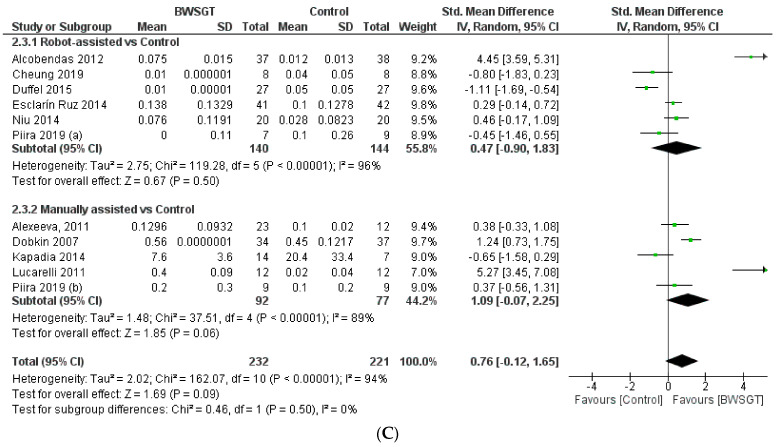
Effectiveness of body weight-supported gait training on gait parameters in patients with motor-incomplete spinal cord injury: (**A**) walking functionality; (**B**) walking endurance; (**C**) walking speed. BWSGT = body weight-supported gait training; SD = standard deviation; IV = inverse variance; CI = confidence interval; Std = standardised. The green point and the horizontal black lines refer to the mean and standard deviation, respectively [23,24,25,27,28,29,30,31,32,33,34,35,36].

Seven randomised controlled trials [23,25,27,29,30,32,36] assessed walking functionality based on mobility independence scale measurements, which are summarised in Figure 3. Overall, the effectiveness of BWSGT was not superior compared to control (n = 356, SMD = 1.25, CI 95%: −0.03 to 2.52) with a high level of heterogeneity (I^2^ = 96%, *p* < 0.00001). None of the randomised controlled trials included in this meta-analysis compared BWSGT to no intervention. The certainty of evidence for this outcome according to GRADE was low in terms of factors to rating down (serious heterogeneity or inconsistency and probable publication bias). In the analysis by subgroups to account for BWSGT, the results showed that RAGT improved walking functionality when compared to the control group (n = 257, SMD = 1.74, CI 95%: 1.09 to 2.39). However, heterogeneity was high (I^2^ = 77%, *p* = 0.002). All studies applying robotic or FES assistance obtained significant improvements in walking functionality, a goal that Dobkin et al. [27] did not achieve. 

When an analysis based on the time since injury was performed (Supplementary Materials), a beneficial effect was observed for BWSGT in chronic patients (n = 62, SMD = 1.82, CI 95%: 0.99 to 2.65) but not in sub-acute patients (n = 294, SMD = 0.76, CI 95%: −1.04 to 2.56). In the analysis by subgroups, RAGT achieved improvements in both subacute (n = 211, SMD = 1.62, CI 95%: 0.78 to 2.46) and chronic (n = 46, SMD = 2.10, CI 95%: 0.59 to 3.61).

#### 3.3.2. Walking Endurance

Seven studies [23,27,28,29,30,33,34] assessed endpoints related to walking endurance using the 6 min walk test. The pooled analysis did not show a superior effect of BWSGT compared to the control (n = 335, MD = 4.87 m; CI 95% = −14.40 to 24.14), with a high level of heterogeneity (I^2^ = 81%, *p* < 0.0001) (Figure 3). None of the studies included in the pooled analysis compared BWSGT to no intervention. The certainty of evidence for this outcome according to GRADE was moderate in terms of rating down (serious heterogeneity or inconsistency). In the analysis by subgroups, RAGT interventions significantly improved walking endurance versus the control (n = 231, MD = 26.59 m, CI 95% = 22.87 to 30.31), with a low heterogeneity value. Only two studies [23,29] that applied RAGT improved walking endurance, unlike manual and functional electrical stimulation assistance, where no trial achieved this objective.

Finally, no significant differences were found globally depending on the time since injury of the participants, although the analysis by subgroups showed an improvement in subacute patients using RAGT (n = 158; MD = 26.85 m, CI 95%: 23.08 to 30.63) (Supplementary Materials).

#### 3.3.3. Walking Speed

Eleven randomised controlled trials [23,24,25,27,28,29,30,31,33,34,35] provided information on walking speed and concluded that BWSGT did not show a superior effect to that of conventional physical therapy or no intervention (n = 453, SMD = 0.66, CI 95% = −0.20 to 1.51), with a high level of heterogeneity (I^2^ = 94%, *p* < 0.0001) (Figure 3). The certainty of evidence for this outcome according to GRADE was low in terms of rating down (serious heterogeneity or inconsistency and probable publication bias). In the analysis by subgroups to account for the intervention, neither RAGT (n = 284, SMD = 0.38, CI 95% = −0.98 to 1.75) nor manually assisted BWSGT (n = 113, SMD = 1.87, CI 95% = −0.04 to 3.79) achieved superior results compared to the control, with high values of heterogeneity (I^2^ = 96% and 92%, respectively). Taken individually, only one study using robotic assistance [23] and two studies using manual assistance [27,31] achieved a significant improvement in gait speed. Similarly, two trials [28,35] compared BWSGT against no intervention and did not observe any improvement.

Finally, in terms of the time since injury, greater effectiveness of the BWSGT in subacute patients (n = 253, SMD = 2.52, CI 95%: 0.64 to 4.40) was observed (Supplementary Materials).

#### 3.3.4. Balance

**Figure 4:**
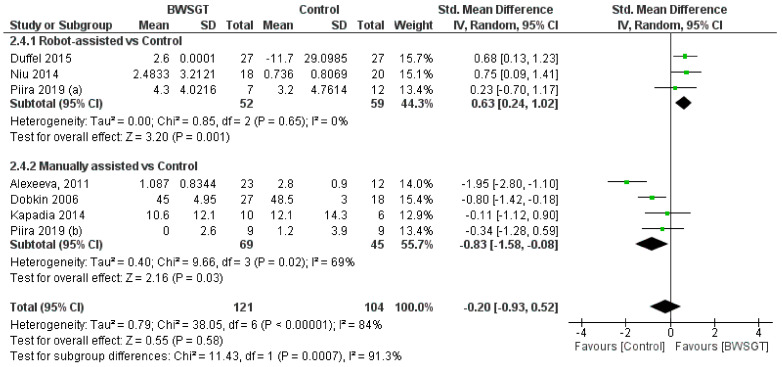
Effectiveness of body weight-supported gait training on balance in patients with motor-incomplete spinal cord injury. BWSGT = body weight-supported gait training; SD = standard deviation; IV = inverse variance; CI = confidence interval; Std = standardised. The green point and the horizontal black lines refer to the mean and standard deviation, respectively. [24,26,28,30,33,34,35].

Seven studies [24,26,28,30,33,34,35] found that the effect of BWSGT on balance was not superior to that of conventional physical therapy or no intervention (n = 225, SMD = −0.20, CI 95% = −0.93 to 0.52), with a high level of heterogeneity (I^2^ = 84%, *p* = 0.00001) (Figure 4). The certainty of evidence for this outcome according to GRADE was low in terms of rating down (serious heterogeneity or inconsistency and probable publication bias). A pooled analysis by subgroups showed a greater effect of RAGT versus the control (n = 111, SMD = 0.63, CI 95% = 0.24 to 1.02). However, in the interventions applying manually assisted BWSGT, a greater effect was observed in the control group (n = 63, SMD = −0.66, CI 95% = −1.18 to −0.14), with I^2^ = 0 in both assistance methods. Two studies [28,35] with a high risk of bias achieved a significant improvement over the control group, although only one of them [35] compared BWSGT to no intervention.

Finally, in terms of time since injury, the overall effect of BWSGT was not superior in subacute or chronic patients (Supplementary Materials). In the analysis by subgroups, however, RAGT obtained significantly superior results versus the control in chronic patients (n = 111, SMD = 0.63, CI 95%: 0.24 to 1.02).

#### 3.3.5. Quality of Life

Two studies [24,26] assessed quality of life, of which only one [24] reported the outcomes, so a pooled analysis could not be conducted. The study found patients to have greater satisfaction in terms of their abilities and well-being (*p* = 0.03 in the intergroup analysis), but no significant results were obtained for general health, mental component of health perception, or vitality.

### 3.4. Publication Bias and Sensitivity Analysis

The visual analysis of the funnel plot (Supplementary Materials) revealed an asymmetry in walking functionality and speed parameters consistent with the presence of publication bias, but not for the walking endurance and balance variables. However, these findings should be interpreted with caution based on the recommendation to not assess the publication bias when including less than ten studies [38]. In the sensitivity analyses, where studies are removed one at a time, the estimates for the pooled effect size were statistically significant for walking functionality when removing the study of Dobkin et al., 2007 [27], and for the walking speed when removing the study of Duffel et al. 2015 [28]. The calculation of the pooled effect size for the balance and walking endurance variables did not show variation when studies were removed from the analysis on a one-by-one basis.

## 4. Discussion

The main outcome of the present meta-analysis showed that BWSGT did not have an overall superior effect on gait parameters and balance compared to conventional physical therapy in patients with motor-iSCI. However, when analysing trials depending on the assistance methods, beneficial effects were observed in RAGT interventions on walking functionality, walking endurance, and balance. To our knowledge, this is the first meta-analysis studying the effectiveness of BWSGT in spinal cord injuries, including only patients with motor-iSCI, and that incorporated balance into the assessed variables.

Previous meta-analyses evaluated the effect of BWSGT in both complete and incomplete spinal-cord-injured patients and found further positive effects compared to conventional physical therapy only when robotic-assisted interventions were included [12,16,17,18,19]. However, systematic reviews without meta-analysis [39,40] that only included patients with motor-iSCI also failed to show that BWSGT was more effective than conventional physical therapy.

In the outcome analysis by subgroups depending on the methods of assistance in the BWSGT interventions, a greater effect of RAGT was observed compared to conventional physical therapy in walking functionality, walking endurance, and balance. The SMD is used to measure the magnitude of the effect, and values of 1.74 and 0.63 are considered to represent a large and moderate effect [41], respectively. However, the improvement in walking endurance could not be considered to reach the minimal clinically important difference, set at 45.8 m [42]. In contrast, conventional physical therapy was superior to manually assisted BWSGT in balance, where it reached a large effect size.

To date, certain reasons appeared to explain why BWSGT did not achieve better results in gait and balance parameters compared to overground gait training without body-weight support in conventional physical therapy programmes. One of these reasons was that overground gait training without body-weight support demands a greater voluntary effort for the initiation and maintenance of gait, thus maximising the supraspinal drive to the spinal locomotor circuitry (a connection that is damaged in spinal-cord-injured subjects and which is essential for the activation of these motor patterns [43,44]). The other reason was that, although both gait training approaches (with and without suspension) seem to share similar kinematic and temporal aspects, there are differences with regard to movements and forces of the lower limb joints and joint power at the knee and hip [45], with BWSGT failing to transfer this training to the motor output that is necessary for overground walking. It seems reasonable to conclude that the overground gait training without the body-weight support task or environment best allows for an individual to learn to generate and control the forces necessary to initiate stepping, to move the body overground, and to practise and improve gait performance [43]. Currently, RAGT offers the possibility of increasing the intensity and total duration of the sessions while maintaining a physiological gait pattern, resulting in increased supraspinal activation and therefore neuroplasticity in patients with motor-iSCI [46,47]. Additionally, RAGT has shown additional advantages, such as an earlier initiation of gait training in severely dependent patients, less effort for the therapists, longer session duration, higher gait intensity, more physiological and reproducible gait patterns, and the ability to measure patients’ performance [48].

Nevertheless, some authors [48,49] suggest that RAGT therapy offers promising effects for functional walking restoration, mainly in subacute and non-chronic spinal cord injury. The results of the present meta-analysis did not fully support this hypothesis, since RAGT was shown to be useful in both subacute (functionality and endurance) and chronic (functionality and balance) patients.

### Limitations

An important limitation of this review was the large heterogeneity in the outcome variables, which lowered the certainty of evidence, and whose high values persisted in the analysis by subgroups. Although determining the factors contributing to this heterogeneity was not possible, the high variability in the demographic characteristics of the subjects, such as the wide range of ages or the different functional capacities of the subjects encompassed within the definition of incomplete spinal cord injury, could have potentially accounted for it. Likewise, the heterogeneity of the protocols in terms of duration and frequency of the sessions or the total duration of the intervention could have strongly contributed to the measurement’s inconsistency. Finally, the ever-present risk of bias in studies must be highlighted, mainly stemming from the lack of blinding of both subjects and the researcher delivering the interventions.

## 5. Conclusions

In conclusion, the current review suggests that, overall, BWSGT is not superior to conventional physiotherapy in gait and balance training. However, BWSGT with robotic assistance does improve walking functionality, walking endurance, and balance, but not walking speed. The certainty of this evidence was graded low-to-moderate, so the results should be handled with caution. Future clinical research should be designed with lower risk of bias in order to improve the certainty of this evidence.

## Figures and Tables

**Table 1:** Characteristics of the included studies.

First Author Year, Country	Participants		Intervention Parameters				Time Points/ Outcome Measures	Losses to Follow-Up
	Age (Mean ± SD)/TSI (Months)/LOL	Randomised (n)/ Analysed (n)	Arms/ Randomised (n) Analysed [n]	Treadmill Speed (m/s)	% BWS	Duration (ss/Week)		
Alcobendas 2012, Spain [23]	45.2 ± 15.5/ <6/above T12	80/75	RAGT (40) [37] CPT (40) [38]	Comfort	Initially 60%; finally goal 25%	5 ss/week 8 weeks	Post-treatment/ WISCI-II, FIM-L, 6 MWT, 10 MWT	5
Alexeeva 2011, USA [24]	38.3 ± 13.5/ >12/above T10	35/35	BWSGT-TRK (14) [14] BWSGT-TM (9) [9] CPT (12) [12]	1.2	30%	3 ss/week 13 weeks	Post-treatment/ WISCI-II, FIM- L, 2 MWT, 10 MWT, SAWS, SF-36	0
Cheung 2019, China [25]	54.3 ± 9.6/ >12/above L2	16/16	RAGT (8) [8] CPT (8) [8]	Comfort	40%	3 ss/week 8 weeks	Post-treatment/ WISCI-II, SCIM-III, Instrumental analysis	0
Dobkin 2006, USA [26]	39.5 ± 15.0/ <6/above L5	59/45	Manual- BWSGT (32) [27] CPT (27) [18]	0.72–1.7	NR	5 ss/week 12 weeks	Post-treatment; FU 6 months/ WISCI-II, BBS	14
Dobkin 2007, USA [27]	NR/ <6/NR	83/78	Manual- BWSGT (43) [39] CPT (40) [39]	NR	NR	5 ss/week 12 weeks	Post-treatment; FU 6 and 12 months/ FIM-L, 6 MWT, 15 MWT	5
Duffel 2015, USA [28]	47.8 ± 13.1/ >12/above T10	56/54	RAGT (27) [27] NI (29) [27]	Comfort and tolerance	Tolerance and comfort	3 ss/week 4 weeks	Post-treatment; FU 1, 2 and 4 weeks/ 6 MWT, 10 MWT, TUG	2
Esclarín 2014, Spain [29]	43.6 ± 12.0/ <6/above L3	88/83	RAGT + CPT (44) [41] CPT (44) [42]	Comfort	Initially 60%; finally goal 25%	5 ss/week 8 weeks	Post-treatment/ WISCI-II, FIM-L, 6 MWT, 10 MWT	5
Kapadia 2014, Canada [30]	56.6 ± 14.0/ >12/above T12	34/27	FES- BWSGT (17) [16] CPT (17) [11]	Natural walking	Minimal to facilitate walking	3 ss/week 16 weeks	Post-treatment; FU 6 and 12 months/ FIM-L, SCIM, 6 MWT, 10 MWT, TUG	7
Lucareli 2011, Brazil [31]	31.4 ± 3.2/ <12/above T12	30/24	Manual BWSGT (15) [12] CPT (15) [12]	0.85–1.25	Initially 40%; ↓10% every 10 ss	2 ss/week 16 weeks	Post-treatment/ Instrumental analysis	6
Mıdık 2020, Turkey [32]	35.4 ± 12.1/ >12/above L3	30/30	RAGT (30) [30] CPT (30) [30]	0.42 to tolerance	Initially 50%; ↓tolerance	3 ss/week 5 weeks	Post-treatment; FU 12 weeks/ WISCI-II, SCIM-III	0
Piira (a) 2019, Norway [33]	55.0 ± 8.0/ >12/above T12	24/19	RAGT (12) [7] CPT (12) [12]	Natural walking	<40%	3 ss/week 24 weeks	Post-treatment/ 6 MWT, 10 MWT, BBS, MFR	5
Piira (b) 2019, Norway [34]	46.0 ± 14.0/ >12/NR	20/18	Manual- BWSGT (10) [9] CPT (10) [9]	0.83–1.39	<40%	10 ss/week 12 weeks	Post-treatment/ 6 MWT, 10 MWT, BBS, MFR	2
Niu 2014, USA [35]	42.2 ± 12.6/ >12/above T10	40/40	RAGT (20) [20] NI (20) [20]	0.94	Minimal to facilitate walking	3 ss/week 4 weeks	Post-treatment/ 10 MWT, TUG	0
Shin 2014, S. Korea [36]	43.2 ± 14.4/ <6/above L5	60/53	RAGT + CPT (30) [27] CPT (30) [26]	0.42	Initially 50%; ↓tolerance	5 ss/week 4 weeks	Post-treatment/ WISCI-II, SCIM-III	7
Varoqui 2014, USA [37]	50.8 ± 2.1/ >12/above T7	30/15	RAGT (15) [15] NI (15) [15]	0.42–0.83	Initially 95%; finally goal 25%	3 ss/week 4 weeks	Post-treatment/ 6 MWT, 10 MWT, TUG	0

SD = standard deviation; TSI = time since injury; LOL = level of lesion; ss = sessions; FU = Follow-up; NR = Not reported; ss: sessions; RAGT: Robotic-assisted gait training; BWSGT: Body weight-supported gait training; TRK: Fixed track; TM: treadmill; CPT: Conventional Physical Therapy; FES = Functional electrical stimulation; NI: no intervention; WISCI-II: walking index for spinal cord injury; FIM-L: Functional Independence Measure- Locomotor; SCIM-III: Spinal Cord Independence Measurement-III; 10 MWT: 10 m walk test; 2 MWT: 2 m walk test; 6 MWT: 6 min walk test; TUG: Timed Up and Go test; BBS: Berg Balance Scale; MFR: Modified Functional Reach test; SAWS: Satisfaction with Abilities and Well-Being Scale; SF-36: Short-Form (36) Health Survey.

## Data Availability

No new data were created or analysed in this study. Data sharing is not applicable to this article.

## Supplementary Materials

The following supporting information can be downloaded at: https://www.mdpi.com/article/10.3390/jcm13041105/s1, electronic search strategy; excluded studies and reasons for exclusion; risk of bias summary; subgroup analysis; funnel plots.
