# Supplementary material for: Effectiveness of Body Weight-Supported Gait Training on Gait and Balance for Motor-Incomplete Spinal Cord Injuries: A Systematic Review with Meta-Analysis
Source: J Clin Med. 2024 Feb 15;13(4):1105. doi: 10.3390/jcm13041105 (PMC10888564; doi:10.3390/jcm13041105)

Supplementary Online Content

Effectiveness of body weight-supported gait training on gait and balance for motor-incomplete spinal cord injuries. A systematic review with meta-analysis.

Supplementary File S1. Electronic search strategy.

Supplementary File S2. Excluded studies and reasons for exclusion.

Supplementary Table S1. Risk of bias summary.

Supplementary Figure S1. Subgroup analysis of effectiveness of BWSGT on walking functionality in patients with iSCI depending on time since injury.

Supplementary Figure S2. Subgroup analysis of effectiveness of BWSGT on walking endurance in patients with iSCI depending on time since injury.

Supplementary Figure S3. Subgroup analysis of effectiveness of BWSGT on walking speed in patients with iSCI depending on time since injury.

Supplementary Figure S4. Subgroup analysis of effectiveness of BWSGT on balance in patients with iSCI depending on time since injury.

Supplementary Figure S5. Funnel plot of the main analysis of BWSGT effectiveness on walking functionality.

Supplementary Figure S6. Funnel plot of the main analysis of BWSGT effectiveness on walking endurance.

Supplementary Figure S7. Funnel plot of the main analysis of BWSGT effectiveness on walking speed.

Supplementary Figure S8. Funnel plot of the main analysis of BWSGT effectiveness on balance.

Supplementary File S1. Electronic search strategy.

PUBMED

#1 paraplegia[Title/Abstract]

#2 quadriplegia[Title/Abstract]

#3 tetraplegria[Title/Abstract]

#4 SCI[Title/Abstract]

#5 spinal injuries[Title/Abstract]

#6 spinal cord injury[MeSH Terms]

#7 #1 OR #2 OR #3 OR #4 OR #5 OR #6

#8 treadmill training[Title/Abstract]

#9 locomotion[Title/Abstract]

#10 robot-assisted[Title/Abstract]

#11 body weigt support*[Title/Abstract]

#12 BWSTT[Title/Abstract]

#13 robotics[MeSH Terms]

#14 lokomat[Title/Abstract]

#15 RAGT OR RALT[Title/Abstract]

#16 #8 OR #9 OR #10 OR #11 OR #12 OR #13 OR #14 OR #15

#17 postural balance[MeSH Terms]

#18 quality of life[MeSH Terms]

#19 gait[Title/Abstract]

#20 #17 OR #18 #19

#21 #7 AND #16 AND #20

WEB OF SCIENCE

#1 TI=(paraplegia) OR AB=(paraplegia)

#2 TI=(tetraplegia) OR AB=(tetraplegia)

#3 TI=(quadriplegia) OR AB=(quadriplegia)

#4 TI=(SCI) OR AB=(SCI)

#5 TI=(spinal cord injur*) OR AB=(spinal cord injur*)

#6 #1 OR #2 OR #3 OR #4 OR #5

#7 TI=(treadmill training) OR AB=(treadmill training)

#8 TI=(locomotion) OR AB=(locomotion)

#9 TI=(robot-assisted) OR AB=(robot-assisted)

#10 TI=(body weight support*) OR AB=(body weight support*)

#11 TI=(BWSTT) OR AB=(BWSTT)

#12 TI=(lokomat) OR AB=(lokomat)

#13 TI=(RAGT OR RALT) OR AB=(RAGT OR RALT)

#14 TI=(robotic) OR AB=(robotic)

#15 #7 OR #8 OR #9 OR #10 OR #11 OR #12 OR #13 OR #14

#16 TI=(balance) OR AB=(balance)

#17 TI=(quality of life) OR AB=(quality of life)

#18 TI=(gait) OR AB=(gait)

#19 #16 OR #17 OR #18

#20 #6 AND #15 AND #19

<u>SCOPUS</u>

#1 TITLE-ABS-KEY (paraplegia)

#2 TITLE-ABS-KEY (quadriplegia)

#3 TITLE-ABS-KEY (tetraplegia)

#4 TITLE-ABS-KEY (SCI)

#5 TITLE-ABS-KEY (spinal injuries)

#6 #1 OR #2 OR #3 OR #4 OR #5

#7 TITLE-ABS-KEY (treadmill training)

#8 TITLE-ABS-KEY (locomotion)

#9 TITLE-ABS-KEY (robot-assisted)

#10 TITLE-ABS-KEY (body weight support*)

#11 TITLE-ABS-KEY (BWSTT)

#12 TITLE-ABS-KEY (robotics)

#13 TITLE-ABS-KEY (lokomat)

#14 TITLE-ABS-KEY (RAGT OR RALT)

#15 #7 OR #8 OR #9 OR #10 OR #11 OR #12 OR #13 OR #14

#16 TITLE-ABS-KEY (Balance)

#17 TITLE-ABS-KEY (quality of life)

#18 TITLE-ABS-KEY (gait)

#19 #16 OR #17 OR #18

#20 #6 AND #15 AND #19

CENTRAL

#1 (paraplegia):ti,ab,kw

#2 (quadriplegia):ti,ab,kw

#3 (tetraplegia):ti,ab,kw

#4 (SCI):ti,ab,kw

#5 (spinal injuries):ti,ab,kw

#6 MeSH descriptor: [Spinal Cord Injuries]

#7 #1 OR #2 OR #3 OR #4 OR #5 OR #6

#8 (treadmill training):ti,ab,kw

#9 (locomotion):ti,ab,kw

#10 (robot-assisted):ti,ab,kw

#11 (body weight support*):ti,ab,kw

#12 (BWSTT):ti,ab,kw

#13 MeSH descriptor: [Robotics]

#14 (lokomat):ti,ab,kw

#15 (RAGT OR RALT):ti,ab,kw

#16 #8 OR #9 OR #10 OR #11 OR #12 OR #13 OR #14 OR #15

#17 MeSH descriptor: [Postural Balance]

#18 MeSH descriptor: [Quality of Life]

#19 (gait):ti,ab,kw

#20 #17 OR #18 #19

#21 #7 AND #16 AND #20

Supplementary File S2. Excluded studies and reasons for exclusion.

Adams MM, Hicks AL (2011) Comparison of the effects of body-weight-supported treadmill training and tilt-table standing on spasticity in individuals with chronic spinal cord injury. J Spinal Cord Med 34:488–494.
Reasons for exclusion: Inappropriate comparator.

Benito J, Kumru H, Murillo N, et al (2012) Motor and gait improvement in patients with incomplete spinal cord injury induced by high-frequency repetitive transcranial magnetic stimulation. Topics in spinal cord injury rehabilitation 18:106–112.
Reasons for exclusion: Inappropriate design.

Carvalho D, de Cássia Zanchetta M, Sereni J, Cliquet A (2005) Metabolic and cardiorespiratory responses of tetraplegic subjects during treadmill walking using neuromuscular electrical stimulation and partial body weight support. Spinal cord 43:400-405.
Reasons for exclusion: Inappropriate outcomes.

Easthope CS, Traini LR, Awai L, et al (2018) Overground walking patterns after chronic incomplete spinal cord injury show distinct response patterns to unloading. J Neuroeng Rehabil: 12;15(1):102.
Reasons for exclusion: Inappropriate outcomes.

Fenuta AM, Hicks AL (2014) Metabolic demand and muscle activation during different forms of bodyweight supported locomotion in men with incomplete SCI. Biomed Res Int 2014:632765.
Reasons for exclusion: Inappropriate comparator and outcomes.

Field-Fote EC, Lindley SD, Sherman AL (2005) Locomotor training approaches for individuals with spinal cord injury: A preliminary report of walking-related outcomes. Journal of Neurologic Physical Therapy 29:127–137.
Reasons for exclusion: Inappropriate comparator.

Field-Fote EC, Roach KE (2011) Influence of a locomotor training approach on walking speed and distance in people with chronic spinal cord injury: a randomized clinical trial. Physical therapy 91:48–60.
Reasons for exclusion: Inappropriate comparator.

Freivogel S, Schmalohr D, Mehrholz J (2009) Improved walking ability and reduced therapeutic stress with an electromechanical gait device. Journal of rehabilitation medicine 41:734-739.
Reasons for exclusion: Inappropriate design.

Gee CM, Sinden AR, Krassioukov AV, Martin Ginis KA (2022) The effects of active upper-limb versus passive lower-limb exercise on quality of life among individuals with motor-complete spinal cord injury. Spinal Cord 60(9):805-811.
Reasons foe exclusion: Inappropriate participants.

Gorman P, Scott W, York H, et al (2016) Robotically assisted treadmill exercise training for improving peak fitness in chronic motor incomplete spinal cord injury: a randomized controlled trial. Journal of spinal cord medicine 39:32-44.
Reasons for exclusion: Inappropriate outcomes.

Houldin A, Luttin K, Lam T (2011) Locomotor adaptations and aftereffects to resistance during walking in individuals with spinal cord injury. J Neurophysiol 106:247–258.
Reasons for exclusion: Inappropriate outcomes.

Knikou M, Mummidisetty CK (2014) Locomotor training improves premotoneuronal control after chronic spinal cord injury. J Neurophysiol 111:2264–2275.
Reasons for exclusion: Inappropriate outcomes.

Kumru H, Benito-Penalva J, Valls-Sole J, et al (2016a) Placebo-controlled study of rTMS combined with Lokomat(®) gait training for treatment in subjects with motor incomplete spinal cord injury. Exp Brain Res 234:3447–3455.
Reasons for exclusion: Inappropriate comparator.

Kumru H, Murillo N, Benito-Penalva J, et al (2016b) Transcranial direct current stimulation is not effective in the motor strength and gait recovery following motor incomplete spinal cord injury during Lokomat(®) gait training. Neurosci Lett 620:143–147.
Reasons for exclusion: Inappropriate comparator.

Labruyere R, van Hedel HJA (2014) Strength training versus robot-assisted gait training after incomplete spinal cord injury: a randomized pilot study in patients depending on walking assistance. J Neuroeng Rehabil 9;11:4.
Reasons for exclusion: Inappropriate design.

Lam T, Pauhl K, Ferguson A, et al (2015) Training with robot-applied resistance in people with motor-incomplete spinal cord injury: Pilot study. J Rehabil Res Dev 52:113–129.
Reasons for exclusion: Inappropriate comparator.

Ma TT, Zhang Q, Zhou TT, Zhang YQ, He Y, Li SJ, Liu QJ (2022) Effects of robotic-assisted gait training on motor function and walking ability in children with thoracolumbar incomplete spinal cord injury. NeuroRehabilitation 51(3):499-508.
Reasons for exclusion: Inappropriate participants.

Moataz M. ES (2019) Influence of percentage of body-weight support on gait in patients with traumatic incomplete spinal cord injury. Egyptian journal of neurology, psychiatry and neurosurgery 55:1-6.
Reasons for exclusion: Inappropriate exposure.

Musselman K, Yang J (2014) Spinal Cord Injury Functional Ambulation Profile: a preliminary look at responsiveness. Physical therapy 94:240-250.
Reasons for exclusion: Inappropriate design.

Nooijen CFJ, Ter Hoeve N, Field-Fote EC (2009) Gait quality is improved by locomotor training in individuals with SCI regardless of training approach. J Neuroeng Rehabil 6:36.
Reasons for exclusion: Inappropriate comparator.

Postans NJ, Hasler JP, Granat MH, Maxwell DJ (2004) Functional electric stimulation to augment partial weight-bearing supported treadmill training for patients with acute incomplete spinal cord injury: A pilot study. Arch Phys Med Rehabil 85:604–610.
Reasons for exclusion: Inappropriate design.

Senthilvelkumar T, Magimairaj H, Fletcher J, et al (2015) Comparison of body weight-supported treadmill training versus body weight-supported overground training in people with incomplete tetraplegia: a pilot randomized trial. Clin Rehabil 29:42–49.
Reasons for exclusion: Inappropriate comparator.

Shin JC, Jeon HR, Kim D, Min WK, Lee JS, Cho SI, Oh DS, Yoo J. Effects of end-effector robot-assisted gait training on gait ability, muscle strength, and balance in patients with spinal cord injury. NeuroRehabilitation. 2023;53(3):335-346.
Reasons for exclusion: Inappropriate intervention.

Wu M, Landry J, Kim J, et al (2016) Repeat Exposure to Leg Swing Perturbations During Treadmill Training Induces Long-Term Retention of Increased Step Length in Human SCI: a Pilot Randomized Controlled Study. Am J Phys Med Rehabil 95:911-920.
Reasons for exclusion: Inappropriate comparator.

Wu M, Landry JM, Schmit BD, et al (2012) Robotic resistance treadmill training improves locomotor function in human spinal cord injury: a pilot study. Arch Phys Med Rehabil 93:782–789.
Reasons for exclusion: Inappropriate comparator.

Yildirim MA, Öneş K, Gökşenoğlu G (2019) Early term effects of robotic assisted gait training on ambulation and functional capacity in patients with spinal cord injury. Turk J Med Sci 49:838–843.
Reasons for exclusion: Inappropriate participants.

Supplementary Table S1. Risk of bias summary.

	Random sequence generation (selection bias)	Allocation concealment (selection bias)	Blinding of participants and personnel (performance bias)	Blinding of outcome assessment (detection bias)	Incomplete outcome data (attrition bias)	Selective reporting (reporting bias)
Alcobendas 2012	+	+	−	+	+	?
Alexeeva, 2011	+	+	−	+	+	?
Cheung 2019	+	+	−	+	+	?
Dobkin 2006	+	?	−	+	+	?
Dobkin 2007	+	?	−	+	+	?
Duffel 2015	?	?	−	−	+	?
Esclarín Ruz 2014	+	+	−	+	+	?
Kapadia 2014	+	+	−	+	+	+
Lucarelli 2011	+	+	−	+	+	+
Midik 2020	+	?	−	−	+	?
Niu 2014	?	?	−	−	−	?
Piira 2019 (a)	+	+	−	+	+	+
Piira 2019 (b)	+	+	−	+	+	+
Shin 2014	?	?	−	−	+	?
Varoqui 2014	?	?	−	−	+	?

Supplementary Figure S1. Subgroup analysis of effectiveness of BWSGT on walking functionality in patients with iSCI depending on time since injury.

A

	BWSGT			Control				Std. Mean Difference
Study or Subgroup	Mean	SD	Total	Mean	SD	Total	Weight	IV, Random, 95% CI
**1.1.1 Subacute (<12m)**								
Alcobendas 2012	10.13	1.77	37	6.42	1.24	38	14.8%	2.41 [1.81, 3.01]
Dobkin 2007	4	0.5	43	4.75	0.25	40	14.9%	-1.86 [-2.38, -1.34]
Esclarín Ruz 2014	7.0237	1.1956	41	5.97	0.923	42	15.0%	0.98 [0.52, 1.44]
Shin 2014	5.25	1.25	27	3.5	1	26	14.8%	1.52 [0.90, 2.14]
**Subtotal (95% CI)**			**148**			**146**	**59.6%**	**0.76 [-1.04, 2.56]**
Heterogeneity: Tau² = 3.29; Chi² = 132.17, df = 3 (P < 0.00001); I² = 98%								
Test for overall effect: Z = 0.83 (P = 0.41)								
**1.1.2 Chronic (>12m)**								
Cheung 2019	1.7	0.68	8	0.1	0.19	8	12.5%	3.03 [1.47, 4.59]
Kapadia 2014	6.4	1.04	10	0.9	5.5	6	13.5%	1.53 [0.35, 2.72]
Midik 2020	3.9	1.1619	15	2.6	0.3873	15	14.4%	1.46 [0.64, 2.28]
**Subtotal (95% CI)**			**33**			**29**	**40.4%**	**1.82 [0.99, 2.65]**
Heterogeneity: Tau² = 0.21; Chi² = 3.20, df = 2 (P = 0.20); I² = 38%								
Test for overall effect: Z = 4.29 (P < 0.0001)								
**Total (95% CI)**			**181**			**175**	**100.0%**	**1.25 [-0.03, 2.52]**
Heterogeneity: Tau² = 2.75; Chi² = 146.00, df = 6 (P < 0.00001); I² = 96%								
Test for overall effect: Z = 1.92 (P = 0.05)								
Test for subgroup differences: Chi² = 1.10, df = 1 (P = 0.29), I² = 9.4%								

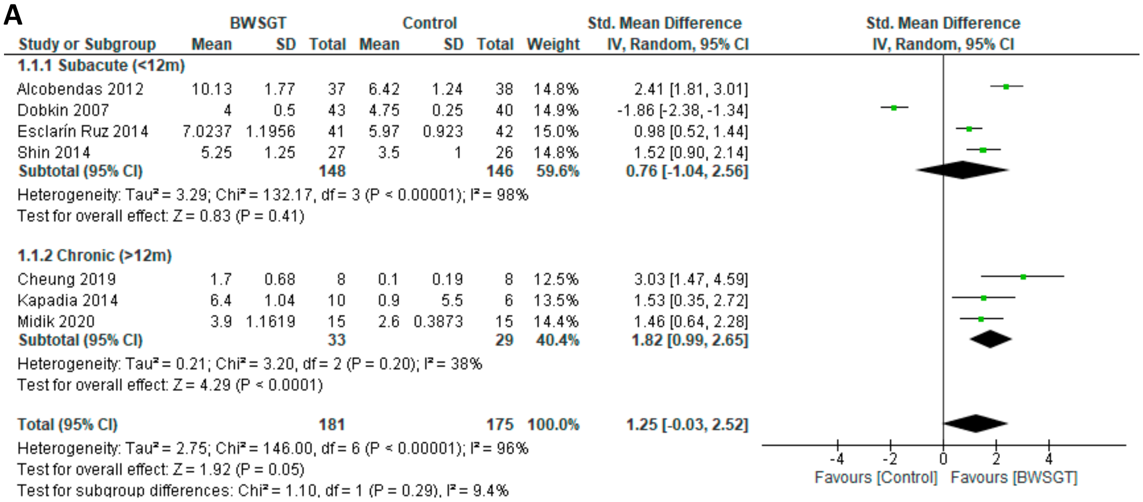

B

	RAGT			Control				Std. Mean Difference
Study or Subgroup	Mean	SD	Total	Mean	SD	Total	Weight	IV, Random, 95% CI
**3.1.1 Subacute**								
Alcobendas 2012	10.13	1.77	37	6.42	1.24	38	22.6%	2.41 [1.81, 3.01]
Esclarín Ruz 2014	7.0237	1.1956	41	5.97	0.923	42	24.7%	0.98 [0.52, 1.44]
Shin 2014	5.25	1.25	27	3.5	1	26	22.4%	1.52 [0.90, 2.14]
**Subtotal (95% CI)**			**105**			**106**	**69.7%**	**1.62 [0.78, 2.46]**
Heterogeneity: Tau² = 0.47; Chi² = 13.77, df = 2 (P = 0.001); I² = 85%								
Test for overall effect: Z = 3.77 (P = 0.0002)								
**3.1.2 Chronic**								
Cheung 2019	1.7	0.68	8	0.1	0.19	8	10.8%	3.03 [1.47, 4.59]
Midik 2020	3.9	1.1619	15	2.6	0.3873	15	19.5%	1.46 [0.64, 2.28]
**Subtotal (95% CI)**			**23**			**23**	**30.3%**	**2.10 [0.59, 3.61]**
Heterogeneity: Tau² = 0.83; Chi² = 3.06, df = 1 (P = 0.08); I² = 67%								
Test for overall effect: Z = 2.72 (P = 0.006)								
**Total (95% CI)**			**128**			**129**	**100.0%**	**1.74 [1.09, 2.39]**
Heterogeneity: Tau² = 0.40; Chi² = 17.36, df = 4 (P = 0.002); I² = 77%								
Test for overall effect: Z = 5.22 (P < 0.00001)								
Test for subgroup differences: Chi² = 0.30, df = 1 (P = 0.59), I² = 0%								

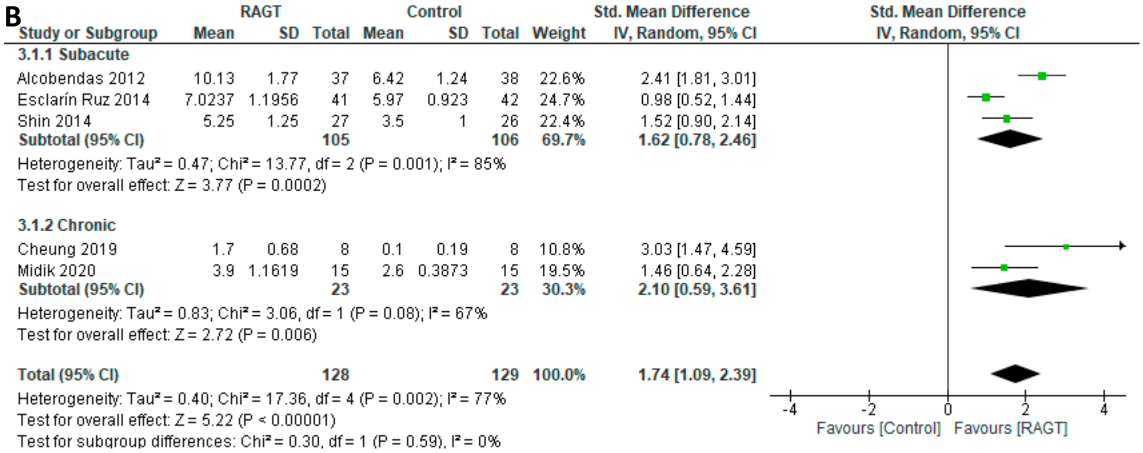

C

	Manually BWSGT			Control				Std. Mean Difference
Study or Subgroup	Mean	SD	Total	Mean	SD	Total	Weight	IV, Random, 95% CI
**3.2.1 Subacute**								
Dobkin 2007	4	0.5	43	4.75	0.25	40	51.3%	-1.86 [-2.38, -1.34]
**Subtotal (95% CI)**			**43**			**40**	**51.3%**	**-1.86 [-2.38, -1.34]**
Heterogeneity: Not applicable								
Test for overall effect: Z = 7.02 (P < 0.00001)								
**3.2.2 Chronic**								
Kapadia 2014	6.4	1.04	10	0.9	5.5	6	48.7%	1.53 [0.35, 2.72]
**Subtotal (95% CI)**			**10**			**6**	**48.7%**	**1.53 [0.35, 2.72]**
Heterogeneity: Not applicable								
Test for overall effect: Z = 2.54 (P = 0.01)								
**Total (95% CI)**			**53**			**46**	**100.0%**	**-0.21 [-3.53, 3.12]**
Heterogeneity: Tau² = 5.54; Chi² = 26.50, df = 1 (P < 0.00001); I² = 96%								
Test for overall effect: Z = 0.12 (P = 0.90)								
Test for subgroup differences: Chi² = 26.50, df = 1 (P < 0.00001), I² = 96.2%								

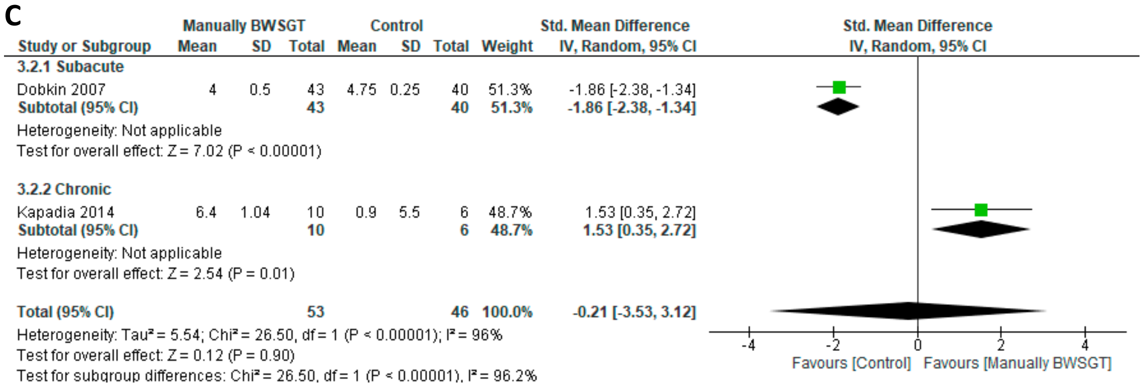

A, BWSGT vs control. B, RAGT vs control. C, Manually assisted BWSGT vs control. BWSGT = body weight supported gait training; SD = standard deviation; IV = inverse variance; CI = confidence interval; Std = standardized

Supplementary Figure S2. Subgroup analysis of effectiveness of BWSGT on walking endurance in patients with iSCI depending on time since injury.

A

Study or Subgroup	BWSGT Mean	BWSGT SD	BWSGT Total	Control Mean	Control SD	Control Total	Weight	Mean Difference IV, Random, 95% CI
**1.2.1 Subacute (<12m)**								
Alcobendas 2012	39.68	9.53	37	12.98	7.28	38	21.2%	26.70 [22.85, 30.55]
Dobkin 2007	218.55	78.1	34	255.58	45.78	36	14.0%	-37.03 [-67.24, -6.82]
Esclarín Ruz 2014	69.8922	49.3295	41	38.715	44.9687	42	17.3%	31.18 [10.86, 51.50]
**Subtotal (95% CI)**			**112**			**116**	**52.4%**	**10.42 [-19.18, 40.03]**
Heterogeneity: Tau^2 = 581.05; Chi^2 = 17.09, df = 2 (P = 0.0002); I^2 = 88%								
Test for overall effect: Z = 0.69 (P = 0.49)								
**1.2.2 Chronic (>12m)**								
Duffel 2015	14.9	39.4908	27	-5.8	42.6084	27	16.7%	20.70 [-1.21, 42.61]
Kapadia 2014	29.2	11	9	51.5	37.9	7	14.4%	-22.30 [-51.28, 6.68]
Piira 2019 (a)	6.6	13.7134	7	23.1	144.1808	12	4.4%	-16.50 [-98.71, 65.71]
Piira 2019 (b)	25.4	40.9	9	29.6	38.2	9	12.1%	-4.20 [-40.76, 32.36]
**Subtotal (95% CI)**			**52**			**55**	**47.6%**	**-1.25 [-25.33, 22.83]**
Heterogeneity: Tau^2 = 274.80; Chi^2 = 5.80, df = 3 (P = 0.12); I^2 = 48%								
Test for overall effect: Z = 0.10 (P = 0.92)								
**Total (95% CI)**			**164**			**171**	**100.0%**	**4.87 [-14.40, 24.14]**
Heterogeneity: Tau^2 = 452.67; Chi^2 = 31.06, df = 6 (P < 0.0001); I^2 = 81%								
Test for overall effect: Z = 0.50 (P = 0.62)								
Test for subgroup differences: Chi^2 = 0.36, df = 1 (P = 0.55), I^2 = 0%								

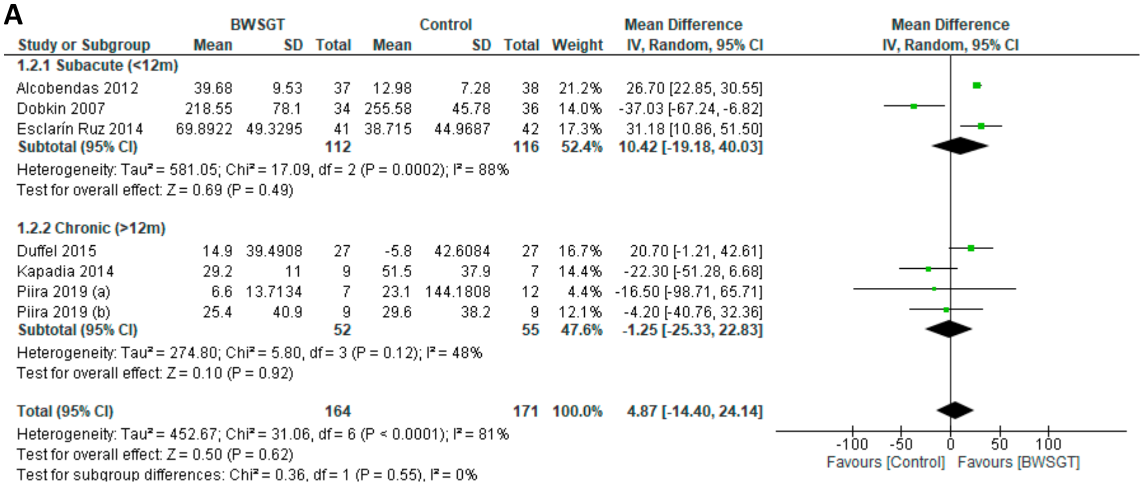

B

Study or Subgroup	RAGT Mean	RAGT SD	RAGT Total	Control Mean	Control SD	Control Total	Weight	Mean Difference IV, Fixed, 95% CI
**3.3.1 Subacute**								
Alcobendas 2012	39.68	9.53	37	12.98	7.28	38	93.6%	26.70 [22.85, 30.55]
Esclarín Ruz 2014	69.8922	49.3295	41	38.715	44.9687	42	3.4%	31.18 [10.86, 51.50]
**Subtotal (95% CI)**			**78**			**80**	**96.9%**	**26.85 [23.08, 30.63]**
Heterogeneity: Chi^2 = 0.18, df = 1 (P = 0.67); I^2 = 0%								
Test for overall effect: Z = 13.93 (P < 0.00001)								
**3.3.2 Chronic**								
Duffel 2015	14.9	39.4908	27	-5.8	42.6084	27	2.9%	20.70 [-1.21, 42.61]
Piira 2019 (a)	6.6	13.7134	7	23.1	144.1808	12	0.2%	-16.50 [-98.71, 65.71]
**Subtotal (95% CI)**			**34**			**39**	**3.1%**	**18.23 [-2.94, 39.41]**
Heterogeneity: Chi^2 = 0.73, df = 1 (P = 0.39); I^2 = 0%								
Test for overall effect: Z = 1.69 (P = 0.09)								
**Total (95% CI)**			**112**			**119**	**100.0%**	**26.59 [22.87, 30.31]**
Heterogeneity: Chi^2 = 1.53, df = 3 (P = 0.67); I^2 = 0%								
Test for overall effect: Z = 14.01 (P < 0.00001)								
Test for subgroup differences: Chi^2 = 0.62, df = 1 (P = 0.43), I^2 = 0%								

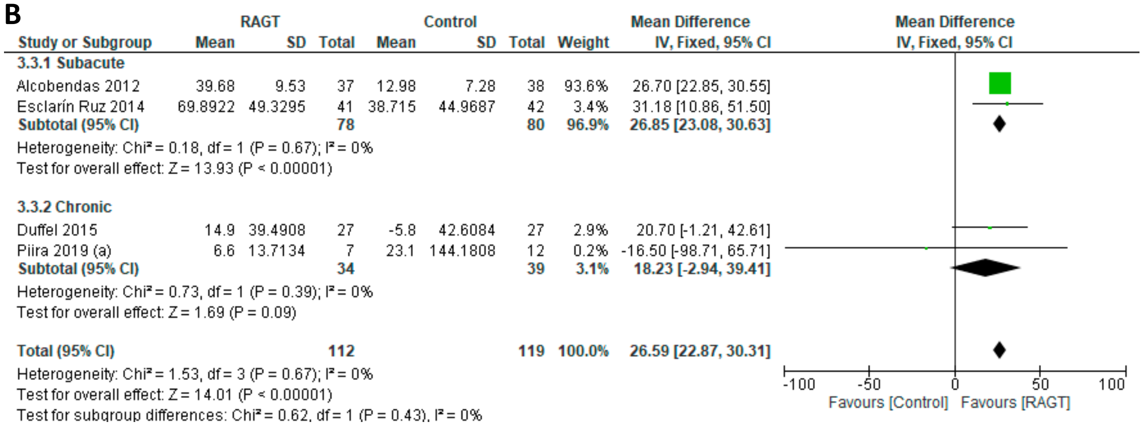

C

Study or Subgroup	Manually BWSGT Mean	Manually BWSGT SD	Manually BWSGT Total	Control Mean	Control SD	Control Total	Weight	Mean Difference IV, Fixed, 95% CI
**3.4.1 Subacute**								
Dobkin 2007	218.55	78.1	34	255.58	45.78	36	36.1%	-37.03 [-67.24, -6.82]
**Subtotal (95% CI)**			**34**			**36**	**36.1%**	**-37.03 [-67.24, -6.82]**
Heterogeneity: Not applicable								
Test for overall effect: Z = 2.40 (P = 0.02)								
**3.4.2 Chronic**								
Kapadia 2014	29.2	11	9	51.5	37.9	7	39.2%	-22.30 [-51.28, 6.68]
Piira 2019 (b)	25.4	40.9	9	29.6	38.2	9	24.7%	-4.20 [-40.76, 32.36]
**Subtotal (95% CI)**			**18**			**16**	**63.9%**	**-15.32 [-38.03, 7.40]**
Heterogeneity: Chi^2 = 0.58, df = 1 (P = 0.45); I^2 = 0%								
Test for overall effect: Z = 1.32 (P = 0.19)								
**Total (95% CI)**			**52**			**52**	**100.0%**	**-23.16 [-41.31, -5.00]**
Heterogeneity: Chi^2 = 1.85, df = 2 (P = 0.40); I^2 = 0%								
Test for overall effect: Z = 2.50 (P = 0.01)								
Test for subgroup differences: Chi^2 = 1.27, df = 1 (P = 0.26), I^2 = 21.1%								

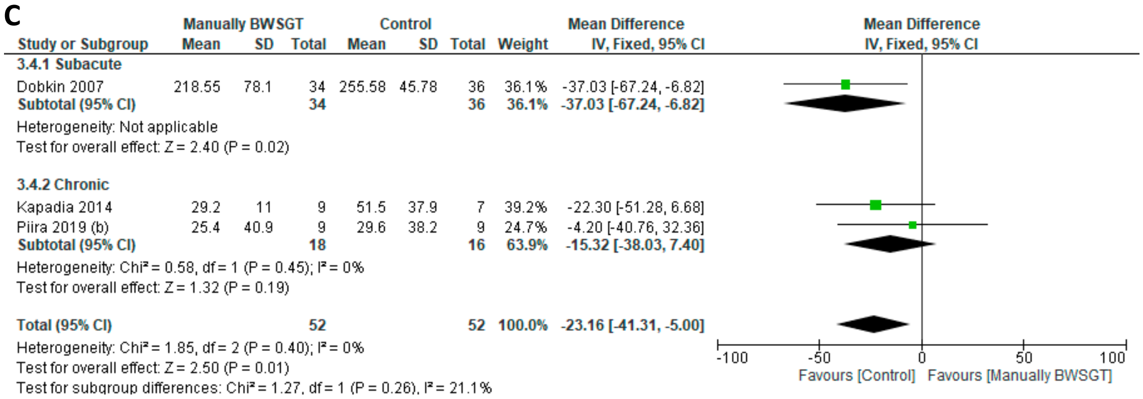

A, BWSGT vs control. B, RAGT vs control. C, Manually assisted BWSGT vs control. BWSGT = body weight supported gait training; SD = standard deviation; IV = inverse variance; CI = confidence interval; Std = standardized

Supplementary Figure S3. Subgroup analysis of effectiveness of BWSGT on walking speed in patients with iSCI depending on time since injury.

A

Study or Subgroup	BWSGT Mean	BWSGT SD	BWSGT Total	Control Mean	Control SD	Control Total	Weight	Std. Mean Difference IV, Random, 95% CI
**1.3.1 Subacute (<12m)**								
Alcobendas 2012	0.075	0.015	37	0.012	0.013	38	9.2%	4.45 [3.59, 5.31]
Dobkin 2007	0.56	0.00001	34	0.45	0.1217	37	9.7%	1.24 [0.73, 1.75]
Esclarín Ruz 2014	0.138	0.1329	41	0.1	0.1278	42	9.8%	0.29 [-0.14, 0.72]
Lucarelli 2011	0.4	0.09	12	0.02	0.04	12	7.0%	5.27 [3.45, 7.08]
**Subtotal (95% CI)**			**124**			**129**	**35.7%**	**2.67 [0.79, 4.56]**
Heterogeneity: Tau^2 = 3.43; Chi^2 = 90.82, df = 3 (P < 0.00001); I^2 = 97%								
Test for overall effect: Z = 2.78 (P = 0.005)								
**1.3.2 Chronic (>12m)**								
Alexeeva, 2011	0.1296	0.0932	23	0.1	0.02	12	9.4%	0.38 [-0.33, 1.08]
Cheung 2019	0.01	0.000001	8	0.04	0.05	8	8.8%	-0.80 [-1.83, 0.23]
Duffel 2015	0.01	0.00001	27	0.05	0.05	27	9.6%	-1.11 [-1.69, -0.54]
Kapadia 2014	7.6	3.6	14	20.4	33.4	7	9.0%	-0.65 [-1.58, 0.29]
Niu 2014	0.076	0.1191	20	0.028	0.0823	20	9.5%	0.46 [-0.17, 1.09]
Piira 2019 (a)	0	0.11	7	0.1	0.26	9	8.9%	-0.45 [-1.46, 0.55]
Piira 2019 (b)	0.2	0.3	9	0.1	0.2	9	9.0%	0.37 [-0.56, 1.31]
**Subtotal (95% CI)**			**108**			**92**	**64.3%**	**-0.25 [-0.80, 0.31]**
Heterogeneity: Tau^2 = 0.38; Chi^2 = 20.24, df = 6 (P = 0.003); I^2 = 70%								
Test for overall effect: Z = 0.87 (P = 0.39)								
**Total (95% CI)**			**232**			**221**	**100.0%**	**0.76 [-0.12, 1.65]**
Heterogeneity: Tau^2 = 2.02; Chi^2 = 162.07, df = 10 (P < 0.00001); I^2 = 94%								
Test for overall effect: Z = 1.69 (P = 0.09)								
Test for subgroup differences: Chi^2 = 8.48, df = 1 (P = 0.004), I^2 = 88.2%								

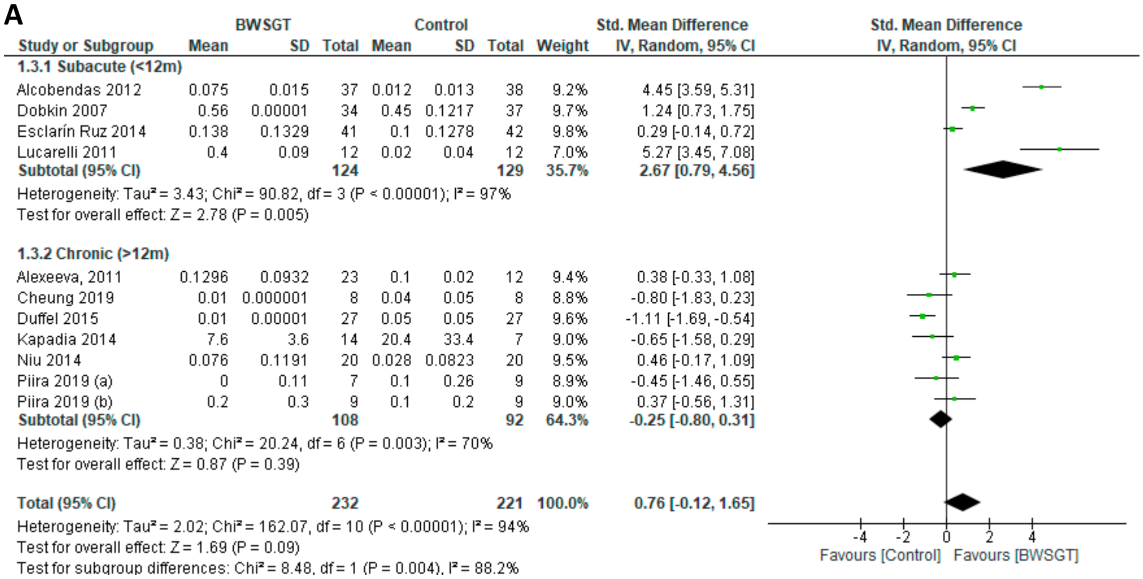

B

Study or Subgroup	RAGT Mean	RAGT SD	RAGT Total	Control Mean	Control SD	Control Total	Weight	Std. Mean Difference IV, Random, 95% CI
**3.5.1 Subacute**								
Alcobendas 2012	0.075	0.015	37	0.012	0.013	38	16.5%	4.45 [3.59, 5.31]
Esclarín Ruz 2014	0.138	0.1329	41	0.1	0.1278	42	17.3%	0.29 [-0.14, 0.72]
**Subtotal (95% CI)**			**78**			**80**	**33.8%**	**2.35 [-1.72, 6.42]**
Heterogeneity: Tau^2 = 8.52; Chi^2 = 71.67, df = 1 (P < 0.00001); I^2 = 99%								
Test for overall effect: Z = 1.13 (P = 0.26)								
**3.5.2 Chronic**								
Cheung 2019	0.01	0.000001	8	0.04	0.05	8	16.0%	-0.80 [-1.83, 0.23]
Duffel 2015	0.01	0.00001	27	0.05	0.05	27	17.1%	-1.11 [-1.69, -0.54]
Niu 2014	0.076	0.1191	20	0.028	0.0823	20	17.0%	0.46 [-0.17, 1.09]
Piira 2019 (a)	0	0.11	7	0.1	0.26	9	16.1%	-0.45 [-1.46, 0.55]
**Subtotal (95% CI)**			**62**			**64**	**66.2%**	**-0.46 [-1.29, 0.36]**
Heterogeneity: Tau^2 = 0.54; Chi^2 = 13.60, df = 3 (P = 0.004); I^2 = 78%								
Test for overall effect: Z = 1.10 (P = 0.27)								
**Total (95% CI)**			**140**			**144**	**100.0%**	**0.47 [-0.90, 1.83]**
Heterogeneity: Tau^2 = 2.75; Chi^2 = 119.28, df = 5 (P < 0.00001); I^2 = 96%								
Test for overall effect: Z = 0.67 (P = 0.50)								
Test for subgroup differences: Chi^2 = 1.76, df = 1 (P = 0.18), I^2 = 43.2%								

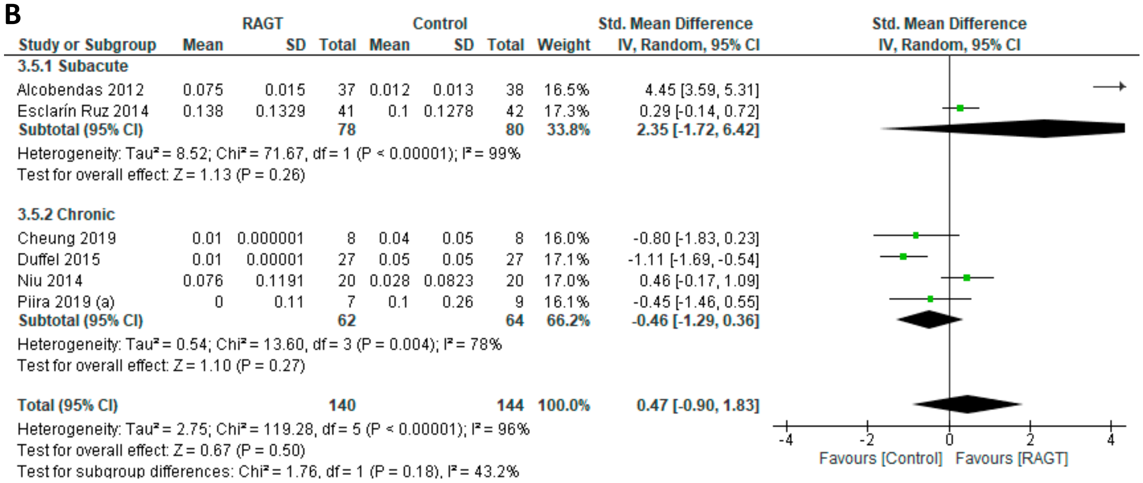

C

Study or Subgroup	Manually BWSGT Mean	Manually BWSGT SD	Manually BWSGT Total	Control Mean	Control SD	Control Total	Weight	Std. Mean Difference IV, Random, 95% CI
**3.6.1 Subacute**								
Dobkin 2007	0.56	0.0000001	34	0.45	0.1217	37	22.5%	1.24 [0.73, 1.75]
Lucarelli 2011	0.4	0.09	12	0.02	0.04	12	14.9%	5.27 [3.45, 7.08]
**Subtotal (95% CI)**			**46**			**49**	**37.4%**	**3.15 [-0.79, 7.10]**
Heterogeneity: Tau^2 = 7.66; Chi^2 = 17.54, df = 1 (P < 0.0001); I^2 = 94%								
Test for overall effect: Z = 1.57 (P = 0.12)								
**3.6.2 Chronic**								
Alexeeva, 2011	0.1296	0.0932	23	0.1	0.02	12	21.7%	0.38 [-0.33, 1.08]
Kapadia 2014	7.6	3.6	14	20.4	33.4	7	20.4%	-0.65 [-1.58, 0.29]
Piira 2019 (b)	0.2	0.3	9	0.1	0.2	9	20.4%	0.37 [-0.56, 1.31]
**Subtotal (95% CI)**			**46**			**28**	**62.6%**	**0.07 [-0.57, 0.71]**
Heterogeneity: Tau^2 = 0.13; Chi^2 = 3.38, df = 2 (P = 0.18); I^2 = 41%								
Test for overall effect: Z = 0.22 (P = 0.82)								
**Total (95% CI)**			**92**			**77**	**100.0%**	**1.09 [-0.07, 2.25]**
Heterogeneity: Tau^2 = 1.48; Chi^2 = 37.51, df = 4 (P < 0.00001); I^2 = 89%								
Test for overall effect: Z = 1.85 (P = 0.06)								
Test for subgroup differences: Chi^2 = 2.29, df = 1 (P = 0.13), I^2 = 56.3%								

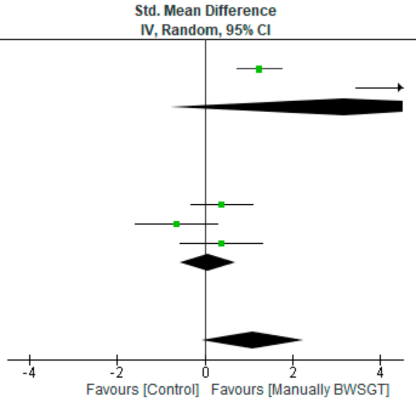

A, BWSGT vs control. B, RAGT vs control. C, Manually assisted BWSGT vs control. BWSGT = body weight supported gait training; SD = standard deviation; IV = inverse variance; CI = confidence interval; Std = standardized

Supplementary Figure S4. Subgroup analysis of effectiveness of BWSGT on balance in patients with iSCI depending on time since injury.

A

Study or Subgroup	BWSGT Mean	BWSGT SD	BWSGT Total	Control Mean	Control SD	Control Total	Weight	Std. Mean Difference IV, Random, 95% CI
**1.4.1 Subacute (<12m)**								
Dobkin 2006	45	4.95	27	48.5	3	18	15.4%	-0.80 [-1.42, -0.18]
**Subtotal (95% CI)**			**27**			**18**	**15.4%**	**-0.80 [-1.42, -0.18]**
Heterogeneity: Not applicable								
Test for overall effect: Z = 2.53 (P = 0.01)								
**1.4.2 Chronic (>12m)**								
Alexeeva, 2011	1.087	0.8344	23	2.8	0.9	12	14.0%	-1.95 [-2.80, -1.10]
Duffel 2015	2.6	0.0001	27	-11.7	29.0985	27	15.7%	0.68 [0.13, 1.23]
Kapadia 2014	10.6	12.1	10	12.1	14.3	6	12.9%	-0.11 [-1.12, 0.90]
Niu 2014	2.4833	3.2121	18	0.736	0.8069	20	15.1%	0.75 [0.09, 1.41]
Piira 2019 (a)	4.3	4.0216	7	3.2	4.7614	12	13.4%	0.23 [-0.70, 1.17]
Piira 2019 (b)	0	2.6	9	1.2	3.9	9	13.4%	-0.34 [-1.28, 0.59]
**Subtotal (95% CI)**			**94**			**86**	**84.6%**	**-0.10 [-0.91, 0.71]**
Heterogeneity: Tau² = 0.85; Chi² = 31.56, df = 5 (P < 0.00001); I² = 84%								
Test for overall effect: Z = 0.24 (P = 0.81)								
**Total (95% CI)**			**121**			**104**	**100.0%**	**-0.20 [-0.93, 0.52]**
Heterogeneity: Tau² = 0.79; Chi² = 38.05, df = 6 (P < 0.00001); I² = 84%								
Test for overall effect: Z = 0.55 (P = 0.58)								
Test for subgroup differences: Chi² = 1.82, df = 1 (P = 0.18), I² = 45.2%								

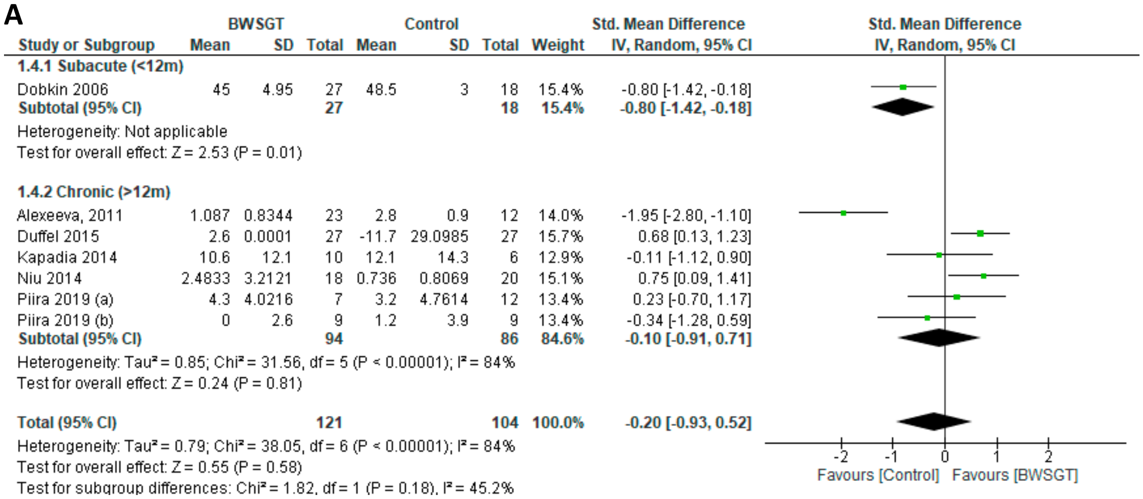

B

Study or Subgroup	RAGT Mean	RAGT SD	RAGT Total	Control Mean	Control SD	Control Total	Weight	Std. Mean Difference IV, Fixed, 95% CI
Duffel 2015	2.6	0.0001	27	-11.7	29.0985	27	49.1%	0.68 [0.13, 1.23]
Niu 2014	2.4833	3.2121	18	0.736	0.8069	20	34.0%	0.75 [0.09, 1.41]
Piira 2019 (a)	4.3	4.0216	7	3.2	4.7614	12	17.0%	0.23 [-0.70, 1.17]
**Total (95% CI)**			**52**			**59**	**100.0%**	**0.63 [0.24, 1.02]**
Heterogeneity: Chi² = 0.85, df = 2 (P = 0.65); I² = 0%								
Test for overall effect: Z = 3.20 (P = 0.001)								

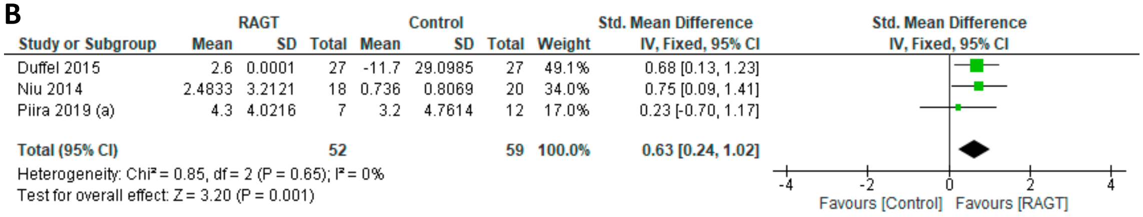

A, BWSGT vs control. B, RAGT vs control. BWSGT = body weight supported gait training; SD = standard deviation; IV = inverse variance; CI = confidence interval; Std = standardized

Supplementary Figure S5. Funnel plot of the main analysis of BWSGT effectiveness on walking functionality.

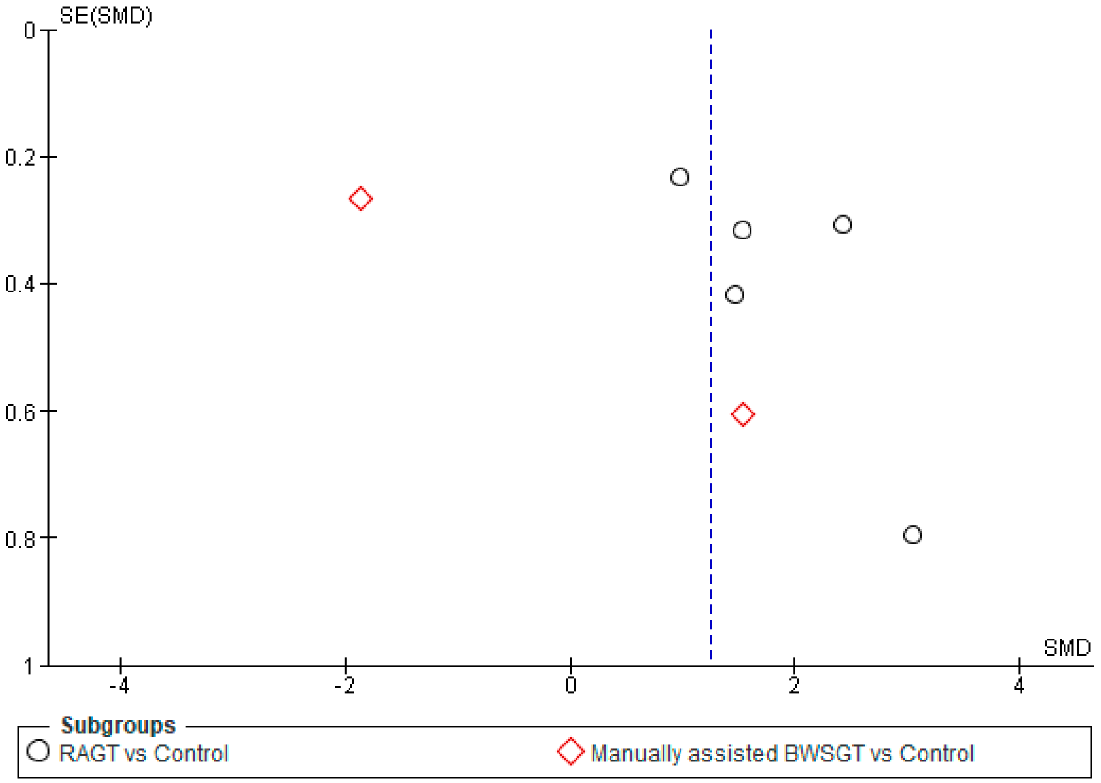

Supplementary Figure S6. Funnel plot of the main analysis of BWSGT effectiveness on walking endurance.

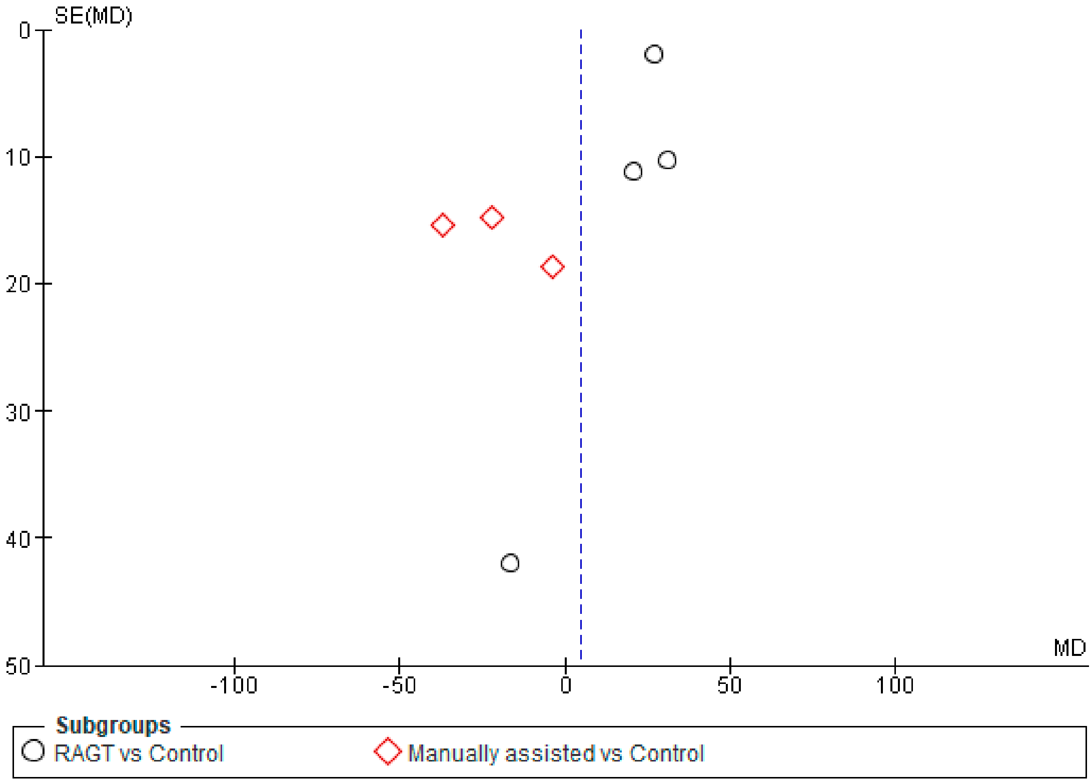

Supplementary Figure S7. Funnel plot of the main analysis of BWSGT effectiveness on walking speed.

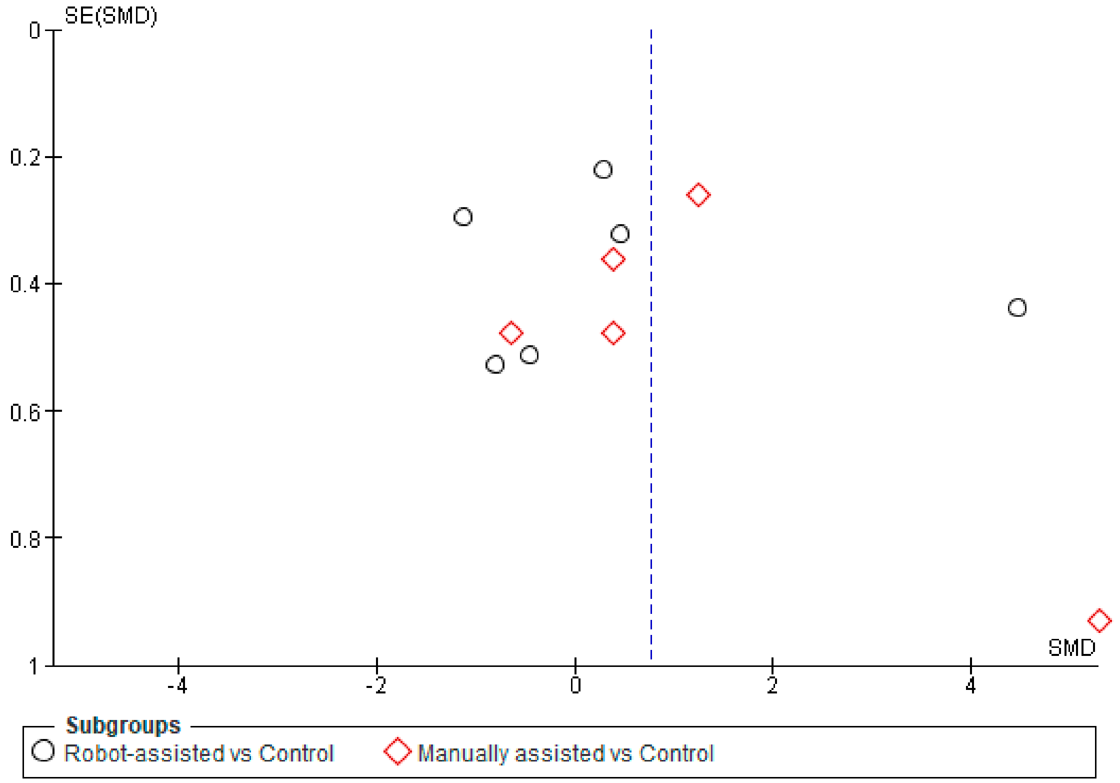

Supplementary Figure S8. Funnel plot of the main analysis of BWSGT effectiveness on balance.

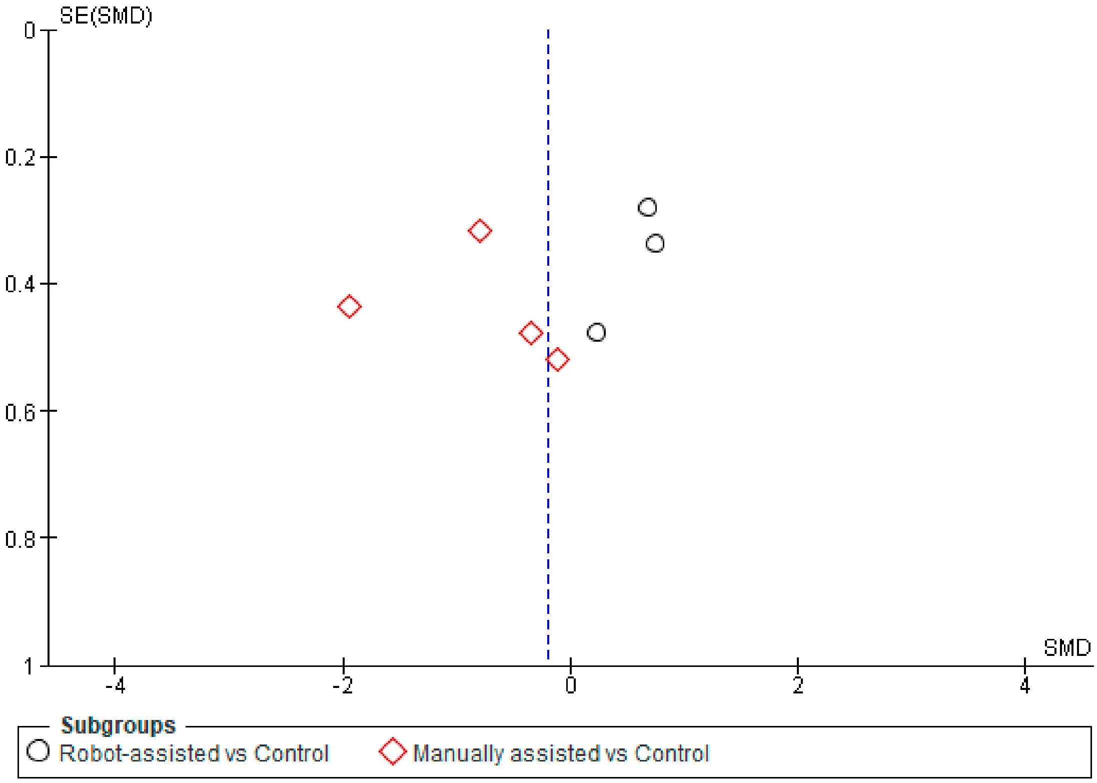

Supplement: Supplementary file 1 [file jcm-13-01105-s001.zip › jcm-2841441-supplementary.pdf]
